# Inhalational Gentamicin Treatment Is Effective Against Pneumonic Plague in a Mouse Model

**DOI:** 10.3389/fmicb.2018.00741

**Published:** 2018-04-24

**Authors:** David Gur, Itai Glinert, Moshe Aftalion, Yaron Vagima, Yinon Levy, Shahar Rotem, Ayelet Zauberman, Avital Tidhar, Arnon Tal, Sharon Maoz, Raphael Ber, Avi Pass, Emanuelle Mamroud

**Affiliations:** ^1^Department of Biochemistry and Molecular Genetics, Israel Institute for Biological Research, Ness Ziona, Israel; ^2^Department of Infectious Diseases, Israel Institute for Biological Research, Ness Ziona, Israel; ^3^Department of Biotechnology, Israel Institute for Biological Research, Ness Ziona, Israel

**Keywords:** *Y. pestis*, plague, infection, gentamicin, tobramycin, mouse model, inhalation, antibiotic treatment

## Abstract

Pneumonic plague is an infectious disease characterized by rapid and fulminant development of acute pneumonia and septicemia that results in death within days of exposure. The causative agent of pneumonic plague, *Yersinia pestis (Y. pestis)*, is a Tier-1 bio-threat agent. Parenteral antibiotic treatment is effective when given within a narrow therapeutic window after symptom onset. However, the non-specific “flu-like” symptoms often lead to delayed diagnosis and therapy. In this study, we evaluated inhalational gentamicin therapy in an infected mouse model as a means to improve antibiotic treatment efficacy. Inhalation is an attractive route for treating lung infections. The advantages include directly dosing the main infection site, the relative accessibility for administration and the lack of extensive enzymatic drug degradation machinery. In this study, we show that inhalational gentamicin treatment administered 24 h post-infection, prior to the appearance of symptoms, protected against lethal intranasal challenge with the fully virulent *Y. pestis* Kimberley53 strain (Kim53). Similarly, a high survival rate was demonstrated in mice treated by inhalation with another aminoglycoside, tobramycin, for which an FDA-approved inhaled formulation is clinically available for cystic fibrosis patients. Inhalational treatment with gentamicin 48 h post-infection (to symptomatic mice) was also successful against a *Y. pestis* challenge dose of 10 i.n.LD_50_. Whole-body imaging using IVIS technology demonstrated that adding inhalational gentamicin to parenteral therapy accelerated the clearance of *Y. pestis* from the lungs of infected animals. This may reduce disease severity and the risk of secondary infections. In conclusion, our data suggest that inhalational therapy with aerosolized gentamicin may be an effective prophylactic treatment against pneumonic plague. We also demonstrate the benefit of combining this treatment with a conventional parenteral treatment against this rapidly progressing infectious disease. We suggest the inhalational administration route as a clinically relevant treatment modality against pneumonic plague and other respiratory bacterial pathogens.

## Introduction

*Yersinia pestis* (*Y. pestis*) is the etiological agent of plague, which has caused millions of deaths in three world pandemics and is an ongoing public health concern in some regions of the world (Pollitzer, 1953; Perry and Fetherston, 1997; Kool, 2005). Recently, an epidemic of pneumonic plague resulting from human-to-human transmission was reported in Madagascar (Tsuzuki et al., 2017). Primary pneumonic plague results from the inhalation of *Y. pestis* droplets or aerosols, leading to a rapidly progressing fatal disease with the capability of spreading from person to person (Pollitzer, 1953; Kool, 2005). These characteristics led to the recognition of *Y. pestis* as a potential bio-threat agent (Inglesby et al., 2000).

Pneumonic plague is an extremely rapidly progressing disease. During the initial 24 h following exposure, the patient is non-infective. In the following several days, symptoms develop, including fever, breathing difficulties, chest pain, pulmonary insufficiency, hemoptysis, and sepsis. Severe bacteremia develops, as well as large numbers of bacteria in the sputum. Pathological analysis in humans reveals acute pneumonia accompanied by massive extracellular bacteremia, severe pulmonary edema and hemorrhaging. Left untreated, death is rapid, usually within 3–4 days of exposure (https://www.cdc.gov). The time of antibiotic therapy initiation is critical, as high mortality rates have been observed if treatment is delayed for longer than 24 h (Inglesby et al., 2000; Kool, 2005).

Effective oral antibiotic treatment for pneumonic plague requires the drug to pass through multiple barriers from the digestive tract to the blood and achieve effective systemic distribution followed by lung uptake. Significant drug losses are incurred by drug metabolism in the digestive tract (Patton and Byron, 2007; Rodvold et al., 2011), imperfect blood absorption, diffusion into irrelevant organs, and drug clearance and detoxification by the kidney and liver. Intravenous (IV) administration directly delivers the drug into circulation but is still subject to clearance mechanisms, limited by the lack of organ specificity and dilution of the drug into the entire circulatory volume. These factors result in a very low fraction of the total administered dose reaching the lungs (Begg et al., 1995). Thus, to achieve effective lung concentrations, the total dose is increased, resulting in adverse effects. Infected lungs may become hypoxic during inflammation, resulting in decreased blood flow, thereby further reducing the circulating drug supply (Rodvold et al., 2011; Sylvester et al., 2012). By targeting the lung directly, these issues are effectively circumvented, ensuring immediate delivery of significant drug doses directly to the target organ. It was previously demonstrated that direct pulmonary delivery of Amikacin increased the lung drug concentration by a factor of over 3,000 compared to IV administration (Begg et al., 1995). Therefore, significant efforts have been invested in recent years to improve pulmonary drug delivery technologies (Begg et al., 1995; Wood, 2011).

In this work, we describe a model system for inhalation treatment of pneumonic plague in mice with aminoglycoside antibiotics, mainly gentamicin, which is recommended by the CDC for the treatment of plague (https://www.cdc.gov).

We demonstrate the high efficacy of post-exposure prophylaxis treatment by inhalation of gentamicin and tobramycin in a mouse model of pneumonic plague. Pulmonary treatment could provide an additional therapeutic option in case of plague outbreaks, where the spread of the disease in the affected population must be rapidly halted. We also show that adding inhalational therapy to conventional parenteral treatment facilitated bacteria lung clearance, which may reduce disease severity and consequent complications as well as the risk of secondary infections.

## Materials and methods

### Ethical statement

This study was performed in accordance with the recommendations for the Care and Use of Laboratory Animals (National Institutes of Health [NIH]). Animal experiments were performed in accordance with Israeli law and were approved by the Institutional Ethics Committee for animal experiments (protocols M-43-15, M-14-16, and M-44-14). During the experiments, the mice were monitored daily. Humane endpoints were used in our survival studies. Mice exhibiting loss of the righting reflex were euthanized by cervical dislocation.

### Animals

Female outbred mice (CD1 from Charles River, UK) weighing 20 g (±4 g) were caged in groups of 3 to 6. Mice were acclimated to their home cage environment at least 3 days before the challenge.

### Antibiotics and treatment protocols

For inhalation treatment, gentamicin sulfate (G-1264, sigma, USA) was dissolved in double distilled water (DDW) at a final concentration of 160 mg/mL, which is equivalent to 100 mg/mL active ingredient. Throughout the paper, gentamicin concentration is presented as the concentration of active ingredient. Gentamicin solution was sprayed using a SideStream nebulizer at a pressure of 3 atm, resulting in a flow rate of 17 L/min. Atomization of the solution was performed at a rate of 0.3 mL/min, generating an aerosol with a concentration of 0.8 mg/L active gentamicin. Assuming an inhalation minute volume of 1 L/kg·min for mice, the 25-g mice would inspire 20 μg/min gentamicin, of which 1.46 μg/min would be deposited in the lower respiratory tract [7.3% alveolar retention for the generated aerosol, according to the aerosol particle deposition rate by size (see below)]. The aerosol was directed into a clear Plexiglas chamber (volume of 14 L) containing the mice. Two running wheels were added to ensure the mice were in motion and not excessively huddled together. The treatment duration was 60 min. Therefore, 87.6 μg of gentamicin was deposited in the alveoli of a 25-g mouse for a dose of 3.5 mg/kg. Tobramycin (PHR-1079, sigma, USA) was used at a concentration of 50 mg/mL, and the inhalation treatment protocol was the same as described above.

For subcutaneous treatment, ciprofloxacin (Ciproxin®, Bayer) was injected in a regimen of 40 mg/kg daily for 5 days, whereas gentamicin was injected in a regimen of 20 mg/kg or 3 mg/mL daily for 5 days.

### Bacteria and infection

The *Y. pestis* strains used in this study included Kimberley53 (Kim53), a fully virulent strain (biovar orientalis) and the bioluminescent Kim53-lux derivative (Vagima et al., 2015; Zauberman et al., 2017). To construct the bioluminescent *Y. pestis* derivative, the plasmid pGEN-luxCDABE (a generous gift from Professor H. Mobley; Lane et al., 2007) was introduced into the *Y. pestis* Kim53 strain by electroporation. Maintenance of the virulence-associated plasmids pMT1, pCD1, and pPCP1 and the *pgm* locus in the Kim53-lux strain was verified by PCR analysis.

Bacterial colonies of *Y. pestis* strains were harvested after growing for 48 h at 28°C on brain heart infusion agar (BHIA) (241830, BD, USA) plates. The bacteria were diluted in heart infusion broth (HIB) (238400, BD, USA) supplemented with 0.2% xylose and 2.5 mM CaCl_2_ (21115, Sigma-Aldrich, Israel) to an OD_660_ of 0.01 and grown for 22 h at 28°C on a shaker (100 rpm). At the end of the incubation period, the cultured bacteria were washed, diluted in saline to the required infectious dose and quantified by plating 0.1 mL of the appropriate dilutions on BHIA plates, incubating for 48 h (at 28°C) and counting colony forming units (CFUs). Prior to infection, the mice were anesthetized with a mixture of 0.5% ketamine HCl and 0.1% xylazine; each mouse was then infected intranasally with 35 μL of the bacterial suspension. The intranasal LD_50_ of the Kim53 strain under these conditions was 2000 CFU. The LD_50_ values were calculated according to the method described by Reed and Muench (1938). To quantify bacterial propagation in the lung and blood, groups of at least four mice were anesthetized, tail vein blood was collected, and the lungs were harvested. Tissue homogenates were prepared in 1 mL of PBS/lung. The bacterial count in tissue homogenates was quantified by plating serial dilutions of homogenate in PBS on BHIA and calculating the CFU/lung or CFU/1 mL of blood.

### Minimally inhibitory concentration (MIC) determination

Antibiotic susceptibility tests and MIC determination were both performed using the Etest® method (Biomerieux, France) on Müeller Hinton agar (MHA) (225250, BD, USA) plates and the standard microdilution method in Müeller Hinton broth (MHB) (212322, BD, USA) according to the CLSI guidelines for *Y. pestis* (CLSI 2015, M45).

### Pharmacokinetic study

The pharmacokinetics of gentamicin was investigated in naïve mice following 1 h of exposure by inhalation, which resulted in a calculated inhaled dose of 3.5 mg/kg. The mice were humanly sacrificed at the indicated time points after antibiotic exposure (3/time point). Serum was collected from blood samples using microtainer® SST® tubes (Beckton Dickinson 365951), and the lungs were aseptically removed, homogenized in 1 mL of PBS (Biological Industries, Beth Haemek, Israel), and centrifuged at 14,000 g for 5 min for supernatant separation. Both sera and lung supernatants were kept frozen at −20°C until determination of the drug levels.

### Pharmacokinetic analysis

Gentamicin concentrations in the serum and lungs were determined by a modified agar diffusion bioassay, using *Staphylococcus aureus* ATCC 29213 as the indicator organism and by comparison to a standard curve of the antibiotic prepared in control mouse sera (limit of detection 1–2 μg/mL). All samples were assayed in triplicate. Pharmacokinetic parameters were derived based on the time vs. tissue concentration profile using the pharmacokinetics software PK Solutions 2.0 (Summit Research Services, Montrose).

### Whole-body imaging using bioluminescence

To evaluate bacterial establishment and proliferation in living mouse tissues in real time, we visualized photon emission using an *in vivo* imaging system (IVIS, Caliper Life Sciences, Hopkinton, MA). We monitored anesthetized mice that were previously infected with 25i.nLD_50_ of Kim53-lux. Daily image acquisition was performed using the following settings: binning of 2 and acquisition times of 1–4 min. The luminescence signals for all images were normalized and reported as photons/second/cm^2^/sr using the Living image® 4 software.

### Development of an inhalational delivery system for antibiotic treatment

#### System description

**Figure 1:**
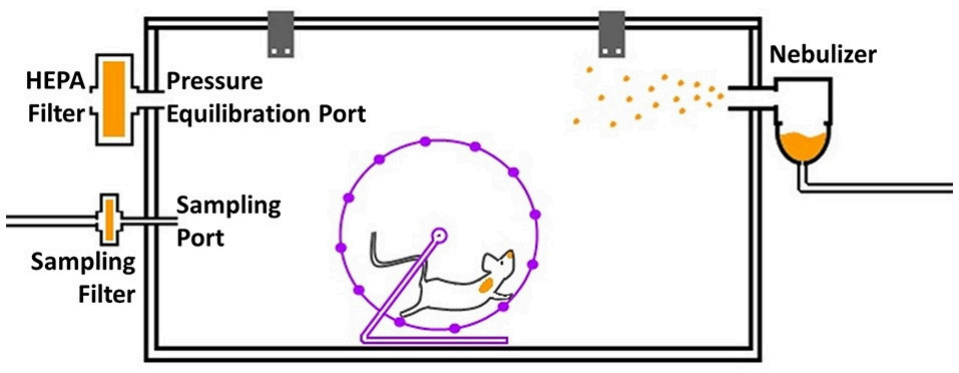
A schematic presentation of the aerosol treatment system. The exposure chamber holds up to 20 mice, and the two running wheels encourage activity and reduce huddling. The nebulizer **(right-hand side)** constantly generates aerosolized antibiotics, which are directed into the chamber. Other openings include a sampling port **(lower left)** connected to a filter and a pressure equilibration port **(upper-left)** connected to a low-resistance HEPA filter.

An induction chamber measuring 39 × 19.5 × 19 cm (internal measurements) with a volume of 14.5 L (VetEquip, CA, USA) was modified by adding several orifices—one 15-mm diameter aerosol entrance (connected to a nebulizer), two 5-mm sampler orifices and a pressure equilibration orifice connected to a low-resistance P3 HEPA filter (RSG-Safety, PW Venlo, The Netherlands). The 47-mm sampling filter housings (Shleicher & Schuell, Dassel, Germany) were fitted to the sampling orifices, and each one was connected to a controlled flow vacuum system. Polycarbonate filters with a pore size of 0.2 μm were used to sample aerosols. This system holds up to 20 free-moving mice per treatment cycle. Two running wheels were added to encourage activity and prevent excessive huddling. A schematic of the system is depicted in Figure 1.

#### Aerosol antibiotic concentration determination

Sampling was performed at a known flow rate of 0.5–1 L/min during aerosol generation. Aerosol was generated using a SideStream nebulizer operating at a flow rate of 17 L/min. Following the sampling period, the filter was removed, and the antibiotics were extracted in 10 mL of DDW. The extracted solution was quantified by a biological assay of bacterial growth inhibition in serial dilutions, similar to an antibiogram test, and compared to the inhibition of a known standard solution. An HPLC measurement was also performed. From these assays, the antibiotic concentration in the aerosol was calculated as follows:

$$\begin{array}{l} Concentration_{Aerosol}=\frac{Concentration_{Extract}\times Volume_{Extract}}{Time_{Sampling}\times Flow_{Sampler}} \end{array}$$

#### Aerosological characterization

Aerosol was sampled at a flow rate of 1 L/min by a seven-stage inertial cascade impactor sampler with a separation range of 0.2–4.5 μm (IN-Tox, New-Mexico, USA). Each stage was extracted and quantified for antibiotic mass as described above. The fractional relative mass at each stage (representing a particular particle size range) was calculated. The median mass of aerosological diameter (MMAD) was calculated using a dedicated algorithm developed in-house. The relative mass of each particle size range was also determined for further calculation of alveolar drug uptake.

#### Calculating the inhaled antibiotic dose

The inhaled dose of antibiotic per mouse was calculated using the aerosol antibiotic concentration (calculated as described above) and the volume inhaled by an average mouse. This has been established (in the literature and by our previous studies) as being ~1 L/kg per minute. The fraction of the antibiotic aerosol that was deposited in the lower respiratory tract of the mice was calculated from the determined fractional relative mass of each particle size range and the known data regarding the size-dependent regional deposition rates for each size range (Raabe et al., 1988). The deposited fraction in our system was calculated to be 7.3% of the total drug concentration in the inhaled aerosol. The overall dose for each drug was controlled by altering the duration of the treatment session.

### Statistical analysis

Statistical significance was determined using the non-parametric Mann-Whitney test. Survival curves were compared using the log-rank test. In all of the analyses, *p*-values equal to 0.05 served as the limit of significance. Calculations were performed using GraphPad Prism software.

## Results

### Development of a gentamicin inhalation therapy system for plague-infected mice

#### General description of the system and setup

As described above, a modified induction chamber was used as the basis for the therapeutic system designed to treat up to 20 mice per treatment cycle. To optimize the treatment and sampling parameters, the correct nebulizer, filter housings and filters as well as the concentration of the nebulized solutions had to be determined. The chamber volume was ~14 L. Aerosol sedimentation was negligible, as the entire volume of the chamber was replaced with freshly generated aerosol every ~50 s.

#### Determination of the nebulization conditions

The nebulization process involved several key parameters, including the type of the nebulizer used, the total airflow applied and the concentration of the nebulized solution. Currently, a major limitation of nebulizers is the relatively low rate of drug delivery. This is a result of low nebulization rates, resulting in the (limited) inhalation of low-concentration aerosols, and compounded by low rates of drug particle deposition in the alveoli. Increasing the concentration of the nebulized solution increases the concentration of the aerosolized drug while simultaneously increasing particle size due to the greater amount of dry mass contained in each generated droplet, which reduces alveolar deposition efficacy, particularly in mice. We tested active gentamicin concentrations of 100 and 230 mg/mL (see Materials and Methods) using a SideStream nebulizer (note: gentamicin concentration throughout the paper is presented as the concentration of active ingredient). The SideStream nebulizer is known for consistent performance and the generation of fine, low-micrometric aerosols, which are suitable for administration in mice. With an MMAD of 1.8 μm when nebulizing a 100 mg/mL gentamicin solution, alveolar uptake in mice was calculated to be 1.46 μL/min/kg. We compared the MMAD and total aerosol concentration to determine the relative mass of each particle size range, which was used to calculate the expected administered doses in mice. We found that at 230 mg/mL, the rate of alveolar uptake was ~3.9%, whereas at 100 mg/mL, the rate was 7.3%; thus, the benefit of the concentration increase was completely offset. We therefore set the maximal concentration as 100 mg/mL for our treatment experiments. Under these conditions, the aerosol concentration was ~0.8 mg/mL gentamicin. Assuming an average mouse respiration rate of 1 L/kg (Raabe et al., 1988) and a mass of 25 g per mouse, the inhaled dose of gentamicin was calculated to be 20 μg/min/mouse. The lower respiratory tract (alveolar) deposition rate for the particle mass and size distribution determined for this aerosol is 7.3%, as stated above, resulting in a total deposited dose of ~1.46 μg/min/mouse. a 60-min exposure is, therefore, 87.6 μg/mouse or ~3.5 mg/kg.

### Pharmacokinetics of gentamicin following inhalation

**Figure 2:**
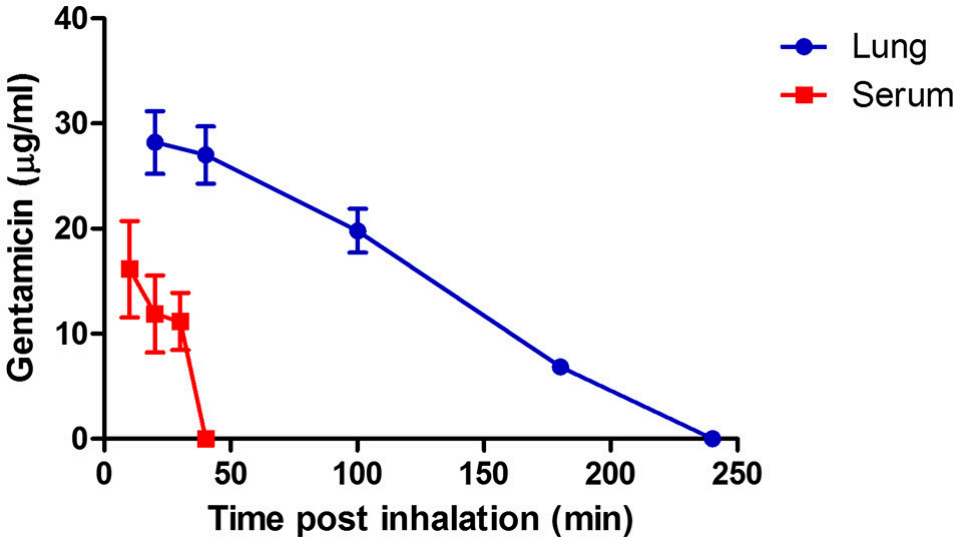
Concentration of gentamicin in the lungs and serum of mice following a single inhalation treatment with gentamicin solution. Average gentamicin concentration in the lungs (blue) and in the serum (red) after 60 min of inhalation of 100 mg/mL gentamicin solution delivered by a SideStream nebulizer (flow rate of 17 L/min). The time points represent min after the end of inhalation. Each data point depicts the mean and the standard error of the mean (SEM) of three individual lung extracts or sera.

The pharmacokinetics of gentamicin in the lungs and serum was investigated in naïve mice following a single inhaled dose of 3.5 mg/kg. Gentamicin concentrations were determined by agar diffusion bioassay. The gentamicin concentrations in the lungs and serum of mice are shown in Figure 2. Pharmacokinetic parameters calculated from these experiments are shown in Table 1. The values of all parameters were found to be higher in the lung than in the serum, which was not surprising considering that the lungs were the target organ of gentamicin via our administration route.

**Table 1:** Pharmacokinetic parameters of gentamicin in the lungs and blood following gentamicin inhalation^a^.

**Organ**	**C_max_ (μg/mL)**	**t_max_ (h)**	**AUC (μg·h/mL)**	**t_1/2_ (h)**
Lungs	28	0.33	53	2.2
Blood	16	0.16	7	0.6

a *Inhalation of 100 mg/mL gentamicin solution delivered by a SideStream nebulizer*.

From the pharmacokinetic data shown in Table 1 and the MIC value of gentamicin for *Y. pestis* Kim53 (0.25 μg/mL, measured by Etest), an area under the curve (AUC)/ minimum inhibitory concentration (MIC) of 212 was achieved in the lungs via inhalation, whereas in the blood stream, the AUC/MIC was 28. The data suggest that inhalation of gentamicin was well-accepted and may be an effective treatment for lung infection with *Y. pestis*.

### Administration of gentamicin via inhalation offers improved therapeutic efficacy

**Figure 3:**
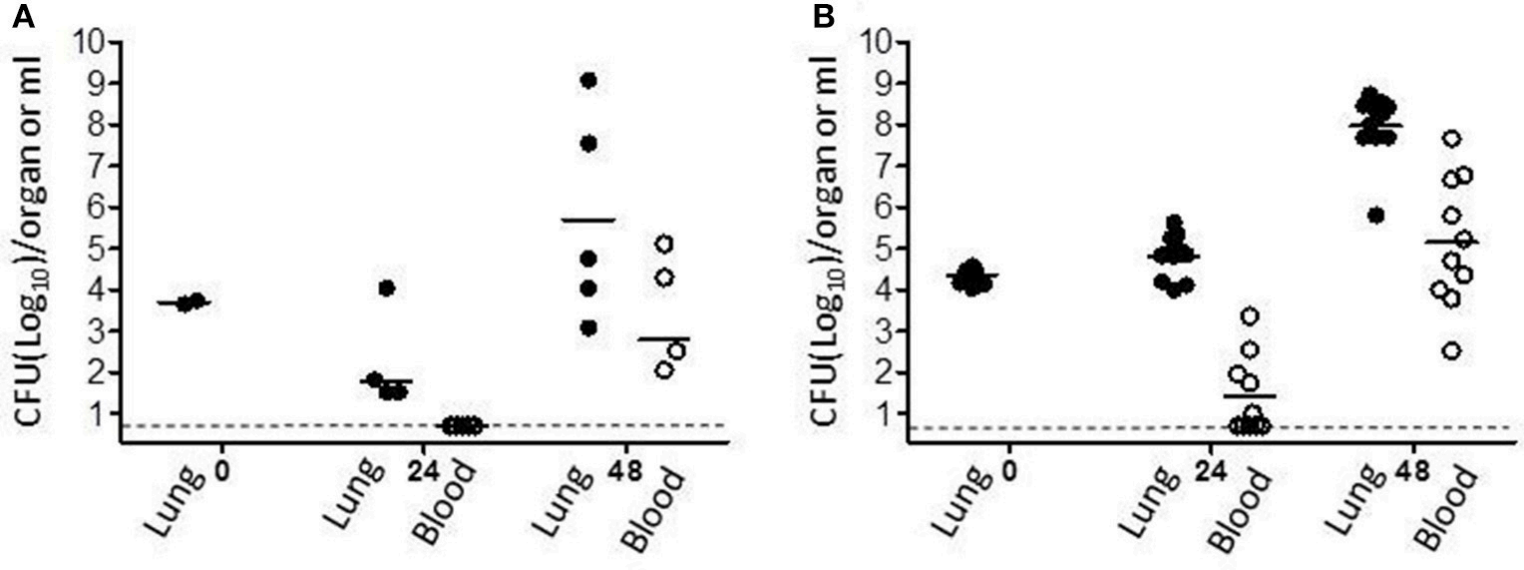
*Y. pestis* loads in mouse organs at the time of antibiotic treatment initiation. Mice were exposed to 10 i.n.LD_50_ **(A)** or 100 i.n.LD_50_ **(B)** of the *Y. pestis* Kim53 strain and sacrificed at the indicated time points. Every point represents the bacterial burden in the lungs (full circles, total CFU/lung) or blood (clear circles, CFU/mL) of an individual mouse. Bacterial burden was quantified by plating the tissue homogenate/blood on BHIA plates in serial dilutions and counting the colonies. The horizontal continuous line represents the geometric mean value. The dashed line marks the level of detection-−5 CFU/mL.

**Figure 4:**
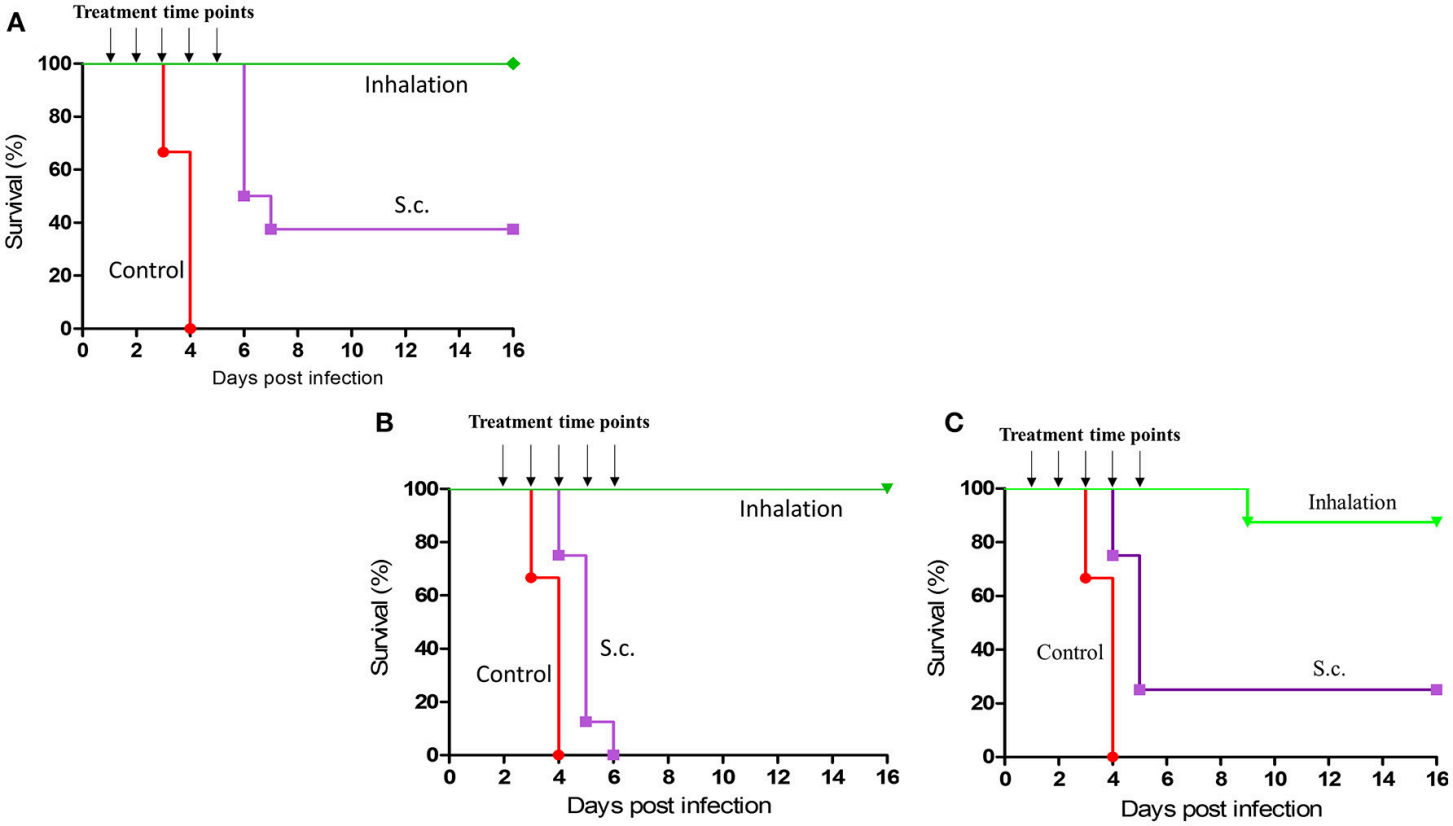
Therapeutic efficacy of early or late initiation of gentamicin inhalation after exposure to 10 i.n. LD_50_ or 100 i.n. LD_50_ of *Y. pestis*. Groups of 8 CD-1 mice were intranasally exposed to 10LD_50_ **(A,B)** or 100LD_50_ **(C)** of *Y. pestis*. Antibiotic treatment with a comparable dose of gentamicin (3.5 mg/kg administered s.c.) was initiated either 24 h after i.n. infection **(A,C)** or 48 h after i.n. infection **(B)** and continued for 5 days as indicated: inhalation (green) and s.c. (purple). Control mice (red) were not treated.

To evaluate the therapeutic efficiency of gentamicin inhalation, we first investigated early initiation of therapy in a group of eight mice 24 h post-infection with a lethal dose of 10 i.n.LD_50_ of the fully virulent *Y. pestis* strain Kim53. At 24 h post-exposure, bacteria were detected in the lungs (average of 10^2^ CFU/organ) but not in the blood (Figure 3A). As shown in Figure 4A, all untreated control mice succumbed to the infection within 4 days, while all mice that were treated with inhalational gentamicin survived the infection.

To compare the efficacy of inhalation treatment to that of the parenteral route, another group of similarly infected mice (*n* = 8) was subcutaneously treated with a comparable low dose of gentamicin (3.5 mg/kg/q24/5 days) starting at 24 h post-infection. This treatment regimen resulted in a survival rate of only 40% (Figure 4A, *p* < 0.001). These data clearly demonstrate the expected advantage of inhalational gentamicin therapy for post-exposure prophylaxis (PEP) from directly targeting the infection site—the lungs. Moreover, when the initiation of treatment was delayed to 48 h after i.n. exposure to 10LD_50_ of *Y. pestis*, the differences between the two administration routes were more substantial (Figure 4B, *p* < 0.001). The main pathophysiological difference was that at this time point, the bacteria have extensively colonized the lungs (an average of 10^6^ CFU/lung) and have already disseminated systemically (Figure 3A). While all the mice treated by inhalational gentamicin at this time point survived the infection, there were no survivors in the group treated parenterally with a comparable dose of gentamicin (Figure 4B).

Inhalational gentamicin still had a therapeutic effect against an increased *Y. pestis* infection dose of 100 i.n.LD_50_, achieving a 90% cure rate when the treatment was started 24 h post-exposure. At this time point, high bacterial loads were observed in the lungs (Figure 3B). Meanwhile, only 25% of the mice that received parenteral treatment survived the infection (Figure 4C, *p* < 0.001). However, at this high infectious dose, both treatment modalities were ineffective when the initiation of treatments was delayed to 48 h post-exposure (data not shown). The inability of the drug treatments to provide protection at this stage of the disease may be due to the high bacterial loads observed in the blood at the time of treatment initiation (Figure 3B) and the insufficient levels of gentamicin in the serum (Figure 2).

### Inhalational gentamicin treatment restricts the growth and dissemination of the virulent *Y. pestis* strain

**Figure 5:**
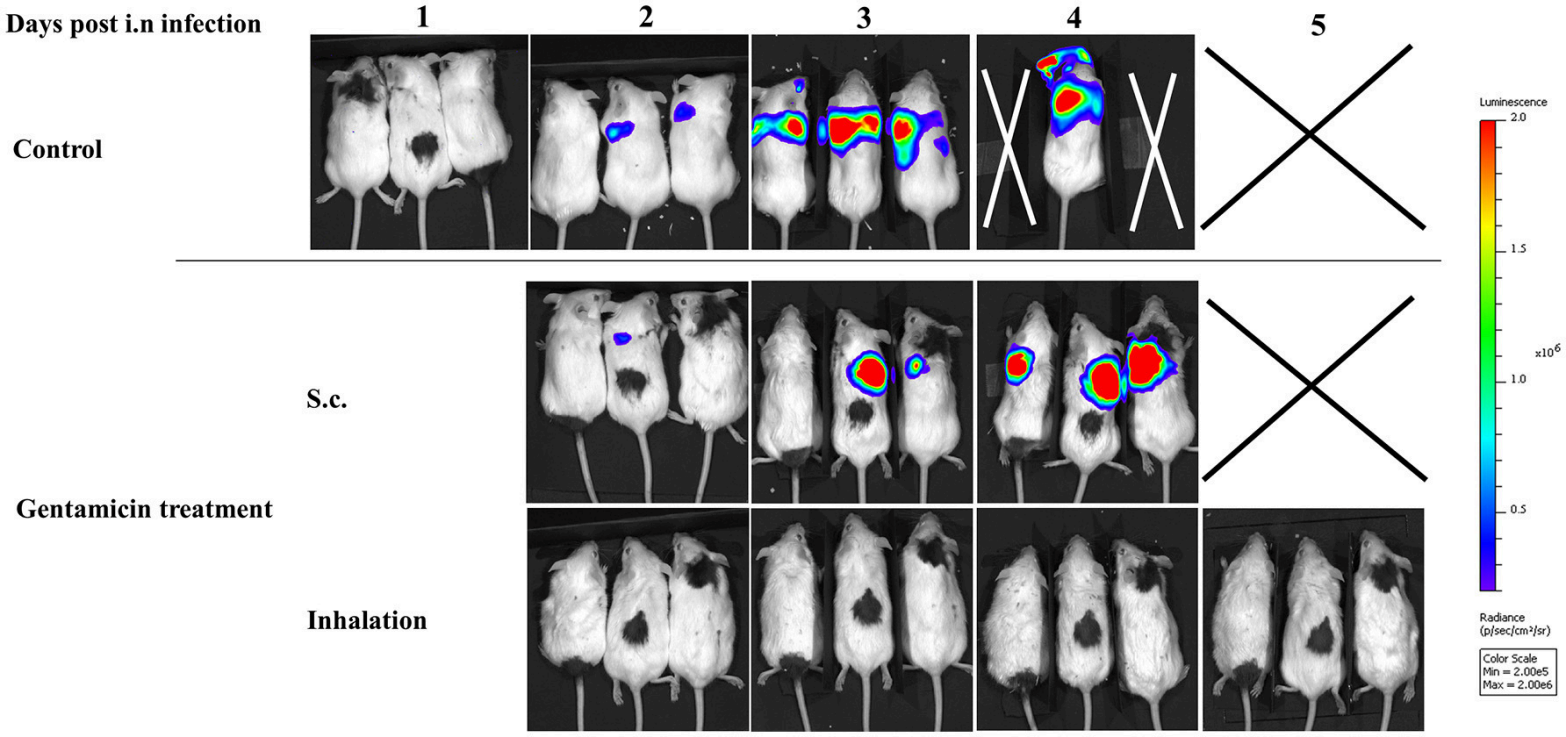
Monitoring bacterial proliferation during pneumonic plague progression by bioluminescence imaging. Mice were treated with a low dose of gentamicin (3.5 mg/kg/q24 s.c. and in 3.5 mg/kg/q24 by inhalation) starting 24 h post-exposure to 25 i.n.LD_50_ of *Y. pestis* Kim53-lux. **(Upper)** s.c. injections, and **(Lower)** inhalation. The mice were marked (black dye) and monitored dorsally in the prone position every 24 h.

To further characterize the effects of inhalational gentamicin treatment on the progression and dissemination of virulent *Y. pestis* bacteria in the host, infected mice were inspected by whole-body imaging using an IVIS. Intranasal infection was performed with a lethal dose of 25 i.n.LD_50_ of the virulent Kim53 derivative expressing a bioluminescence cassette (Kim53-lux). Individual animals were monitored daily for bioluminescence emission, which reflected the dissemination of bacteria. As depicted in the upper panel of Figure 5, in the non-treated group, a signal marking the presence of at least 5 × 10^5^ bacteria (the detection limit) was visible in the lungs at day 2 post-infection. In the following days, the bioluminescent *Y. pestis* expanded in the lungs and disseminated until day 5, at which time all untreated mice were dead. Conversely, mice treated by inhalational gentamicin starting at 24 h post-exposure did not exhibit any bioluminescence signals throughout the experiment (Figure 5, lower panel), and no mortality was observed in this group. Meanwhile, an increase in bioluminescence signals was observed in the lungs of mice treated parenterally with a comparable dose of gentamicin (Figure 5, middle panel). All mice in this treatment group died by day 5 post-infection. These results further demonstrate the advantage of prompt and direct administration of antibiotics to the target organ of the pathogen.

### Combination of inhaled and parenteral antibiotic treatments

While our inhalational gentamicin therapy (3.5 mg/kg/q24 h for 5 days) was unable to protect the mice at late stages of disease progression due to dissemination of the bacteria to the blood, full protection was achieved at this progressive stage of infection by conventional parenteral treatment of mice with a high dose of gentamicin (20 mg/kg/q24 h/s.c. for 5 days, Figure S1). Interestingly, treatment was successful although the drug did not reach the lungs of the mice (Table 2), suggesting that protection against the bacteria was mediated by the immune system in the lungs while the antibiotics addressed systemic bacteria outside the lungs.

**Table 2:** Pharmacokinetic parameters of gentamicin following a single subcutaneous injection at 20 mg/kg.

**Organ**	**C_max_ (μg/mL)**	**t_max_ (h)**	**AUC (μg·h/mL)**	**t_1/2_ (h)**
Blood	53	0.25	25	0.4
Lungs	0	0	0	0

**Figure 6:**
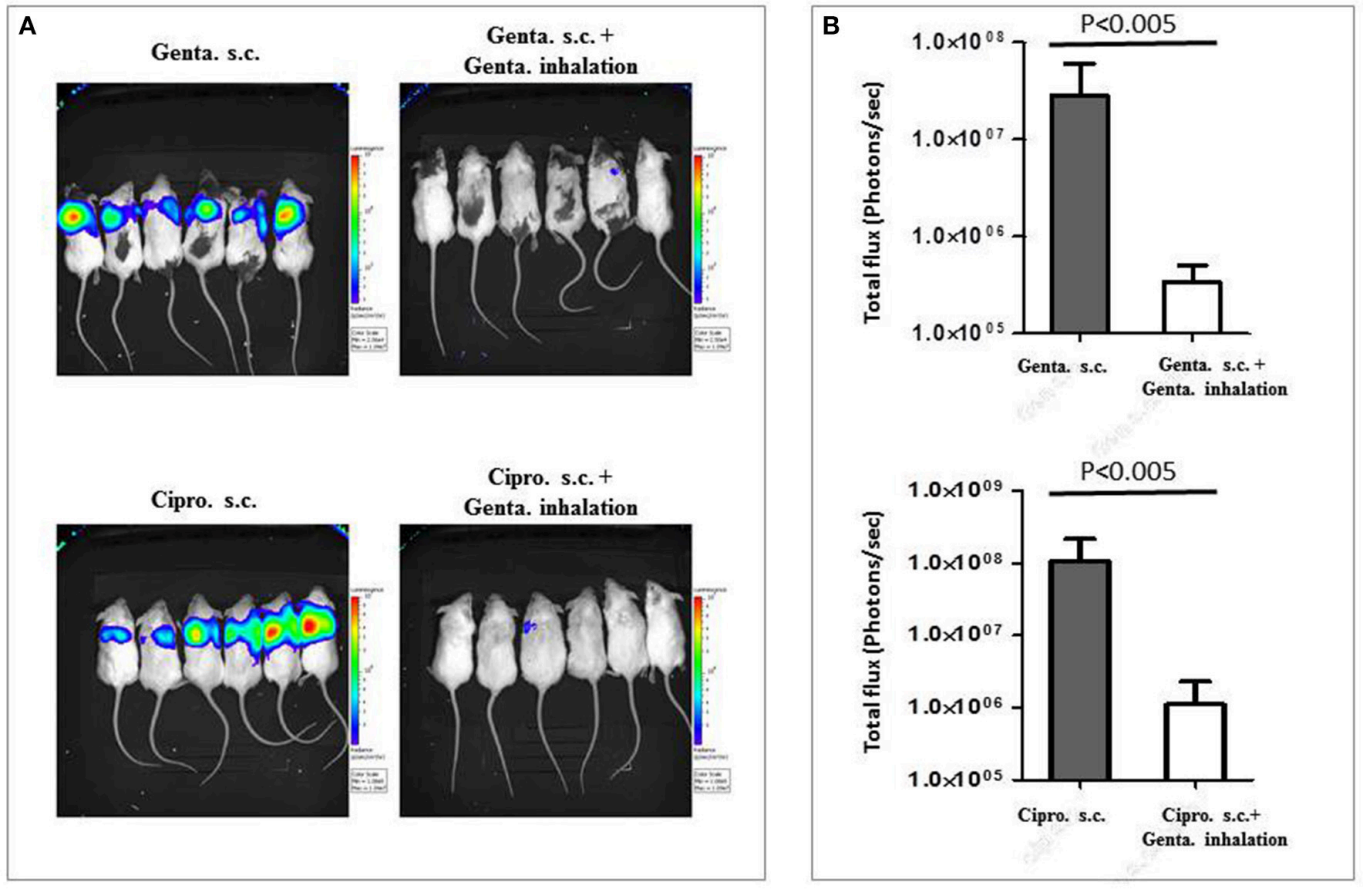
Combining gentamicin inhalation with parenteral treatment against severe pneumonic plague. Mice were treated s.c. with the indicated antibiotics (gentamicin or ciprofloxacin) for 5 days starting 48 h after i.n. exposure to 25LD_50_ of the bioluminescent *Y. pestis* Kim53-lux strain (20 mg/kg/q24 h). Mice in the right column were additionally treated with inhalational gentamicin starting at 48 h after i.n. infection, as described above (3.5 mg/kg/q24 h). The images present the condition of the mice 72 h post-exposure (24 h after the 1st treatment) **(A)**. Light intensity around lungs of treated mice (Gentamicin-Genta., Ciprofloxacin-Cipro.), was quantified by ROI analysis **(B)**. Statistical significance was determined using the non-parametric Mann-Whitney test.

The effective reduction of lung bacterial loads during inhalation treatment with gentamicin (Figure 5) and the high local concentration of gentamicin in the lungs during inhalational therapy (Figure 2) raised the possibility of improving bacteria clearance from the lungs by parenteral treatment combined with inhalational therapy. To test this hypothesis, we exposed groups of six mice to 25 i.n.LD_50_ of the bioluminescent *Y. pestis* strain Kim53-lux. The first group (Figure 6A, upper panel, left) was treated with gentamicin only via the s.c. route starting 48 h post-exposure (20 mg/kg/q24). The second group (Figure 6A, upper panel, right) was treated parenterally, as described for the first group, and also received inhalational gentamicin (3.5 mg/kg/q24/5 days). The bioluminescence signals, which reflect bacterial load, were visualized by IVIS 72 h post-infection (24 h after 1st treatment). As shown in the upper panels of Figures 6A,B, the addition of inhalational gentamicin dramatically reduced the bacterial load in the lungs. In mice that received only parenteral treatment, the observable reduction of bacterial load began 4 days after infection, and the infection was not resolved until day 6 (data not shown). Improvement of bacterial clearance from the lungs by combining inhalational gentamicin therapy and protective parenteral treatment with ciprofloxacin (Figure S1) was also demonstrated (40 mg/kg, Figures 6A,B, lower panels). These findings suggest that although parenteral treatment protects against severe pneumonic plague, parenteral treatment alone does not clear bacteria from the lungs until nearly 1 week after infection. In contrast, the addition of gentamicin inhalation treatment results in rapid clearance of bacteria from the lungs, potentially improving the condition of the lungs after recovery and reducing the risk of secondary infections.

### Effective treatment by inhalation of tobramycin

**Figure 7:**
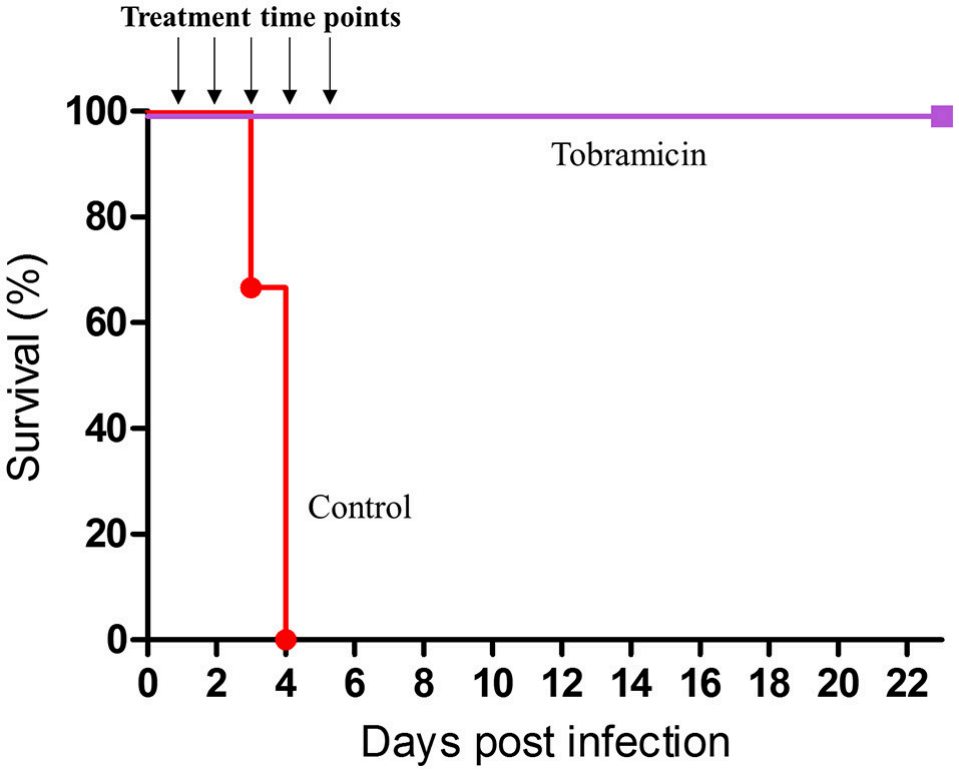
Therapeutic efficacy of inhaled tobramycin. Groups of 8 CD-1 mice were exposed intranasally to 15LD_50_ of *Y. pestis* Kim53. Inhalational treatment with tobramycin solution (50 mg/mL 1 h daily, (deposition of 1.7 mg/kg in the lungs) was initiated 24 h after i.n. infection and continued for 5 days. Control mice were not treated.

Tobramycin, like gentamicin, is an aminoglycoside approved by the FDA for treatment of cystic fibrosis (CF) patients by inhalation (Konstan et al., 2011; Harrison et al., 2014). Since inhalational tobramycin exists as a shelf product (Tobi), we examined its therapeutic efficiency against pneumonic plague. Twenty-four hours post-exposure to 10 i.n.LD_50_ of *Y. pestis*, we initiated a 5-day treatment of 60 min inhalation of 50 mg/mL tobramycin solution per day (1.5 mg/kg/q24). Similar to gentamicin inhalation, tobramycin rescued 100% of the infected animals, while the non-treated mice succumbed to the infection by day 4 (Figure 7). These findings indicate that inhalational tobramycin may also be considered as an early post-exposure treatment against pneumonic plague.

## Discussion

The underlying principle of inhalational antibiotic therapy for lung infections is extremely direct and straightforward: delivering the drug directly to the desired action site achieves a high local concentration with a low systemic dose, thus reducing adverse effects (Cipolla et al., 2016). This principle is of great importance in scenarios of mass exposure of the populace to bio-terror agents that are mainly lethally infective by inhalation (anthrax, tularemia, and plague, among others). These situations require a rapid and widespread therapeutic with which to treat those suspected of exposure soon after its occurrence, prior to disease onset. Once the disease is symptomatic, treatment efficacy declines rapidly, and the burden on public health services increases with the need to hospitalize and intensely care for the sick. This is the reason why most so-called “prophylactic treatment” drugs are available as oral treatment regimens. Using cheap disposable inhalers that can deliver targeted antibiotic doses with greater therapeutic efficacy may reduce the overall treatment duration. Furthermore, the need for trained medical personnel, such as nurses and doctors, whom are required to administer drugs intravenously, will be reduced. Thus, an inhalational regimen may prove to be both clinically viable and cost effective. In conjunction with mass prophylactic treatment, inhalational antibiotics may augment and complement the efficacy and safety of standard intensive-care antibiotic treatment practices.

This study evaluated gentamicin, a CDC-recommended antibiotic for pneumonic plague, in a mouse model. Pneumonic plague is a fatal disease that leads to rapid deterioration if left untreated. The virulence strategies of *Y. pestis*, the causative agent of plague, allow the pathogen to escape the immune system, proliferate at the infection site—the lungs—and disseminate to other internal organs and blood (Lathem et al., 2005; Vagima et al., 2012; Yang et al., 2017).

Using a relatively simple system built in-house, we successfully applied inhalational gentamicin treatment to pneumonic plague-infected mice. In a recently published study (Hamblin et al., 2017), a similar approach was applied to administer two liposomal formulations of ciprofloxacin by inhalation to mice infected with pneumonic plague. It is worth noting that mice are a challenging model for inhalation studies due to the stringent restraints regarding the inhaled particle size, which is significantly smaller than the inhalable size for humans (Raabe et al., 1988). We therefore anticipate that this treatment modality will be less technically challenging for human application, as evident in the use of inhalation therapy for CF patients (Dalhoff, 2014). Our system could treat 20 non-anesthetized mice per treatment cycle, delivering ~3.5 mg/kg gentamicin directly to the lower respiratory tract in an hour-long treatment session.

Our pharmacokinetic analysis demonstrated that while the drug was indeed slowly cleared from the respiratory tract, effective doses at well above the MIC were detected for at least 3.5 h after administration (Table 1, Figure 2). The full parenteral dose of 20 mg/kg did not result in detectable doses of antibiotics in the lungs of treated mice (Table 2).

The therapeutic efficacy of these treatment modalities were then tested in mice infected intranasally with 10 or 100LD_50_ of the *Y. pestis* strain Kim53, with the initiation of treatment at either 24 or 48 h after infection. In the group infected with 10LD_50_ of *Y. pestis*, inhalation of gentamicin at both early and delayed treatment initiation time points was effective (Figures 4A,B). Treatment efficacy despite delayed treatment initiation is extremely important, as the infection has already begun to spread systemically by this time point (Figure 3A). Thus, this finding indicated that even after systemic spread of the disease, direct treatment of the major site of infection and the consequent dissemination of antibiotics into the blood stream are sufficient for effective clearance and recovery. Moreover, monitoring the spread of disease in animals during the course of treatment with a luminescence-based IVIS imaging system clearly demonstrated that inhalation treatment controlled the *Y. pestis* infection (25 i.n.LD_50_) better than systemic treatment with a comparable dose of parenterally administered gentamicin (Figure 5).

When a higher *Y. pestis* infection dose was used (100LD_50_), inhalation treatment was still effective when initiated 24 h after infection (Figure 4). This again demonstrates the potential for treating exposed individuals by inhalation treatment, even if the projected exposure dose is high. However, delaying treatment initiation to 48 h following exposure to this infection dose resulted in treatment failure, indicating that pulmonary treatment with such a low total dose cannot sufficiently address extensive, systemic bacterial infections. When combined with effective parenteral gentamicin therapy (Figure S1), inhalation of gentamicin improved the clearance of bacteria from the lungs and rapidly reduced the bacterial burden in infected mice (Figure 6). Similar results (Figure 6) were achieved when the inhaled gentamicin treatment was combined with effective parenteral ciprofloxacin treatment (Figure S1). These findings may have clinical significance, as inhalation therapy may help shorten treatment times and reduce disease-induced lung damage, thereby potentially reducing morbidity if not mortality. Another important effect is that rapid reduction in lung bacterial burden may reduce the risk of secondary infections.

Finally, the effect of tobramycin on plague-infected mice was also evaluated due to the availability of this drug in an FDA-approved inhalable formulation. Early post-exposure treatment with tobramycin was shown to be effective against pneumonic plague (Figure 7).

In conclusion, we provided proof of concept that inhalational aminoglycoside therapy is an effective post-exposure therapeutic tool against pneumonic plague. Although inhalers capable of administering sufficient therapeutic doses of antibiotics are not yet available, they can be rapidly developed, and we are sure to see such inhalers before long. In summary, antibiotic administration via inhalation seems to be a clinically relevant treatment modality against pneumonic plague and other respiratory bacterial pathogens.

## Author contributions

DG, IG, AP, RB, and EM conceived and designed the study. DG, IG, MA, YV, YL, SR, AZ, ATi, ATa, SM, RB, AP, and EM conducted the experiments. DG, IG, AP, SR, RB, and EM analyzed the data. DG, IG, and EM wrote the paper.

### Conflict of interest statement

The authors declare that the research was conducted in the absence of any commercial or financial relationships that could be construed as a potential conflict of interest.

## Supplementary material

Figure S1 Therapeutic efficacy of parenteral treatment with Gentamicin and ciprofloxacin. Groups of 8 CD-1 mice were exposed intranasally to 100LD_50_ of *Y. pestis* Kim53. Mice were treated s.c. with 20 mg/kg/q24 h gentamicin (blue) or with 40 mg/kg/q24 h ciprofloxacin (green) for 5 days starting 48 h after exposure to *Y. pestis*. Control mice were not treated (red).
