# Supplementary figures and images for: Inhalational Gentamicin Treatment Is Effective Against Pneumonic Plague in a Mouse Model
Source: Front Microbiol. 2018 Apr 24;9:741. doi: 10.3389/fmicb.2018.00741 (PMC5928325; doi:10.3389/fmicb.2018.00741)

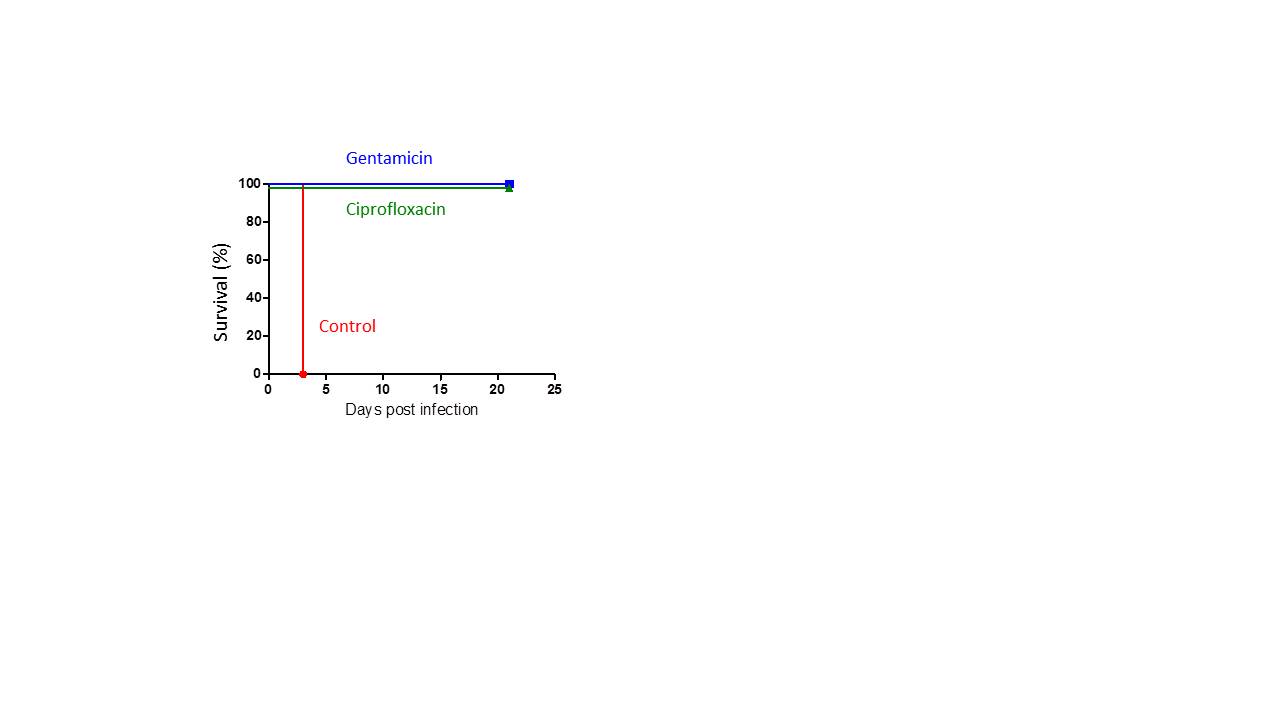

Supplement: Figure S1 — Therapeutic efficacy of parenteral treatment with Gentamicin and ciprofloxacin. Groups of 8 CD-1 mice were exposed intranasally to 100LD50 of Y. pestis Kim53. Mice were treated s.c. with 20 mg/kg/q24 h gentamicin (blue) or with 40 mg/kg/q24 h ciprofloxacin (green) for 5 days starting 48 h after exposure to Y. pestis. Control mice were not treated (red). [file Image_1.jpeg]
